# Smartphone-derived mobility metrics identify distinct recovery phenotypes following ACL reconstruction: associations with function, pain, and psychological health

**DOI:** 10.1186/s43019-026-00353-1

**Published:** 2026-10-26

**Authors:** Praveen Kumar Kandakurti, Ghada Mohamed Koura, Ravi Shankar Reddy, Saleh Kardm, Ajay Prashad Gautam, Ahmed Mohamed Fathi Elshiwi, Zeinab A. Ali, Rahaf Saad Alali, Ahmed M. El Melhat

**Affiliations:** 1 College of Health Sciences, Gulf Medical University, Ajman, United Arab Emirates; 2 Physical Therapy Program, Department of Medical Rehabilitation Sciences, College of Applied Medical Sciences, King Khalid University, Abha, Saudi Arabia; 3 Department of Orthopedic Surgery, College of Medicine, Najran University, Najran, Saudi Arabia; 4 Department of Physical Therapy, Saudi German Hospital, Aseer, Saudi Arabia; 5 Department of Physical Therapy and Health Rehabilitation, College of Applied Medical Sciences, Jouf University, Sakakah, Saudi Arabia; 6 Department of Physical Therapy for Surgery, Faculty of Physical Therapy, Cairo University, Cairo, Egypt; 7 Department of Physical Therapy for Musculoskeletal Disorders and its surgery, Faculty of Physical Therapy, Cairo University, Giza, Egypt; 8 Department of Physical Therapy, Faculty of Health Sciences, Beirut Arab University, Beirut, Lebanon

**Keywords:** Anterior cruciate ligament reconstruction, Smartphone, Digital phenotyping, Functional recovery, Patient-reported outcomes, Wearable sensors

## Abstract

**Background:**

Recovery after anterior cruciate ligament (ACL) reconstruction is typically monitored through patient-reported outcomes and periodic clinical assessments, which may not fully capture real-world functional performance. Smartphone-derived mobility data provide a passive and objective approach for evaluating daily activity and recovery.

**Purpose:**

To examine associations between smartphone-derived mobility metrics and recovery outcomes in adults 3–12 months after primary ACL reconstruction and to identify distinct recovery phenotypes using mobility-based clustering.

**Methods:**

This cross-sectional study included 120 adults who were 3–12 months post-primary ACL reconstruction and had at least 14 days of retrospective smartphone mobility data. Mobility variables included daily step count, activity volume and intensity (e.g., active minutes and walking bout duration), sedentary behavior, and walking-related metrics. Outcomes included pain (Numeric Rating Scale [NRS]), functional status (Knee Injury and Osteoarthritis Outcome Score [KOOS] Pain, Activities of Daily Living, and Sport/Recreation subscales), knee-related quality of life (ACL Quality of Life questionnaire [ACL-QoL]), and psychological measures including the Pain Catastrophizing Scale (PCS), Patient Health Questionnaire-9 (PHQ-9), and Generalized Anxiety Disorder-7 (GAD-7). Associations were evaluated using Pearson correlation and multivariable linear regression analyses. Recovery phenotypes were identified using unsupervised *k*-means clustering of mobility metrics.

**Results:**

Higher daily step counts were independently associated with better KOOS-ADL scores (*β* = 0.48, *p* < 0.001), higher ACL-QoL scores (*β* = 0.45, *p* < 0.001), and lower pain intensity (*β* = − 0.39, *p* = 0.001). Three recovery phenotypes emerged: a high-activity/low-pain phenotype with superior function and minimal psychological distress, a moderate-activity/moderate-pain phenotype with intermediate outcomes, and a low-activity/high-pain phenotype characterized by reduced mobility, poorer function, greater pain, and higher pain catastrophizing. Significant gradients were observed across phenotypes for pain (NRS: 1.72–5.01), function (KOOS-Pain: 88.94–62.37), and pain catastrophizing (PCS: 8.52–24.91; all *p* < 0.001).

**Conclusions:**

Smartphone-derived mobility metrics were significantly associated with recovery outcomes following ACL reconstruction. Mobility-based clustering identified distinct recovery phenotypes associated with meaningful differences in pain, function, and pain catastrophizing, highlighting the potential of passive mobility monitoring to support personalized rehabilitation strategies. However, given the variability in evaluation periods and uncontrolled surgical factors, future research employing standardized, long-term follow-ups is warranted.

## Introduction

Anterior cruciate ligament (ACL) reconstruction is a commonly performed orthopedic procedure aimed at restoring knee stability and enabling return to activity following ligament rupture [1]. Despite advancements in surgical techniques and rehabilitation protocols, a substantial proportion of individuals experience delayed or incomplete recovery, with persistent pain, impaired function, or psychological barriers limiting return to preinjury levels of activity [2]. Traditionally, recovery is monitored through episodic clinical assessments and patient-reported outcome measures (PROMs), which, while informative, are subject to recall bias, interpretive variability, and limited ecological validity [3]. These approaches are typically conducted in structured clinical environments or rely on retrospective self-report, which may not accurately capture habitual, unsupervised movement behavior in daily life. As a result, they may underrepresent fluctuations in activity patterns, contextual influences, and real-world functional performance that occur outside controlled testing settings [3]. In an era of personalized medicine, there is increasing recognition of the need for continuous, objective, and contextually relevant metrics to complement PROMs and better capture functional recovery in real-world settings [4].

Recent developments in digital health have enabled the use of smartphone-based data to continuously and passively track physical activity [5]. Smartphones, widely adopted across age groups, integrate sensors and platforms—such as Apple Health, Google Fit, and Samsung Health—that automatically record step counts, active minutes, and mobility-related metrics [5]. Prior studies have shown that activity levels measured by wearable or smartphone sensors are associated with postoperative outcomes in orthopedic populations [6, 7]. Wu et al. [8] demonstrated correlations between step count and KOOS scores after knee arthroplasty, while Ullrich et al. [9] reported that real-world gait metrics predicted fall risk in older adults. In ACL populations, however, much of the existing literature has focused on laboratory-based motion analysis or structured functional tests, with limited attention to passively acquired, ecologically valid mobility data [10]. Furthermore, prior research has typically examined average activity levels in isolation, without considering temporal patterns such as variability, sedentary duration, or consistency of engagement, which may offer additional insights into the quality and sustainability of recovery [11].

There remains a critical need to determine whether smartphone-derived mobility metrics—collected unobtrusively in daily life—can meaningfully reflect self-reported recovery outcomes following ACL reconstruction. While PROMs remain the standard for assessing subjective pain and functional outcomes, they may not fully capture day-to-day behavior or adherence to rehabilitation goals [12]. Passive mobility tracking has the potential to provide a continuous, objective complement to PROMs, enabling a more holistic understanding of recovery [13]. Importantly, whether such data can be used to identify distinct recovery phenotypes that align with clinical needs or psychological burden remains unclear. Understanding these phenotypes could guide stratified rehabilitation approaches, allowing clinicians to tailor interventions based on real-world mobility profiles rather than relying solely on periodic self-report or performance-based testing. The present study aimed to (1) evaluate the association between smartphone-derived mobility metrics and standard PROMs reflecting pain, function, and quality of life among individuals 3–12 months post-ACL reconstruction, and (2) identify distinct recovery phenotypes using unsupervised clustering of mobility data, and compare PROMs, psychological burden, and rehabilitation needs across these groups. We hypothesized that smartphone-derived mobility metrics would be significantly associated with patient-reported recovery outcomes and that unsupervised clustering of mobility patterns would identify clinically meaningful recovery phenotypes with distinct profiles of pain, function, psychological burden, and rehabilitation needs.

## Material and methods

### Study design, ethics, and settings

This cross-sectional study was conducted between January and September 2024 at the Orthopedic and Sports Rehabilitation Clinic, Department of Physical Therapy, XXXX University. Participant recruitment and study assessments were performed during the same study window described above, and all smartphone mobility data were retrospectively extracted from the 30 days preceding each participant’s study visit. Ethical approval was obtained from the Institutional Review Board of XXXX University (APPROVAL NUMBER: XXXX), and all participants provided written informed consent before enrollment. The study adhered strictly to the ethical standards outlined in the Declaration of Helsinki.

### Participants

Participants were consecutively recruited from an outpatient orthopedic and sports rehabilitation clinic between January and September 2024. Eligible individuals were adults aged 18–45 years who had undergone primary unilateral ACL reconstruction 3–12 months before enrollment, a period selected to capture the subacute-to-late rehabilitation phase characterized by substantial variability in functional recovery, symptom burden, and psychological readiness. All ACL injuries resulted from sports-related or traumatic mechanisms and were confirmed using established clinical criteria, including positive Lachman and pivot-shift tests and preoperative magnetic resonance imaging (MRI) findings. Participants underwent standardized arthroscopic single-bundle reconstruction performed by one of three fellowship-trained orthopedic knee surgeons, using a uniform institutional protocol emphasizing anatomic tunnel placement, predefined graft-selection criteria, and consistent fixation techniques. Reconstruction was performed using either BTB or hamstring tendon autografts, and operative reports were reviewed to document graft characteristics, fixation methods, and concomitant intra-articular procedures, including meniscal repair, partial meniscectomy, and treatment of focal chondral lesions. Revision ACL reconstruction and complex multiligament injuries were excluded.

**Fig. 1:**
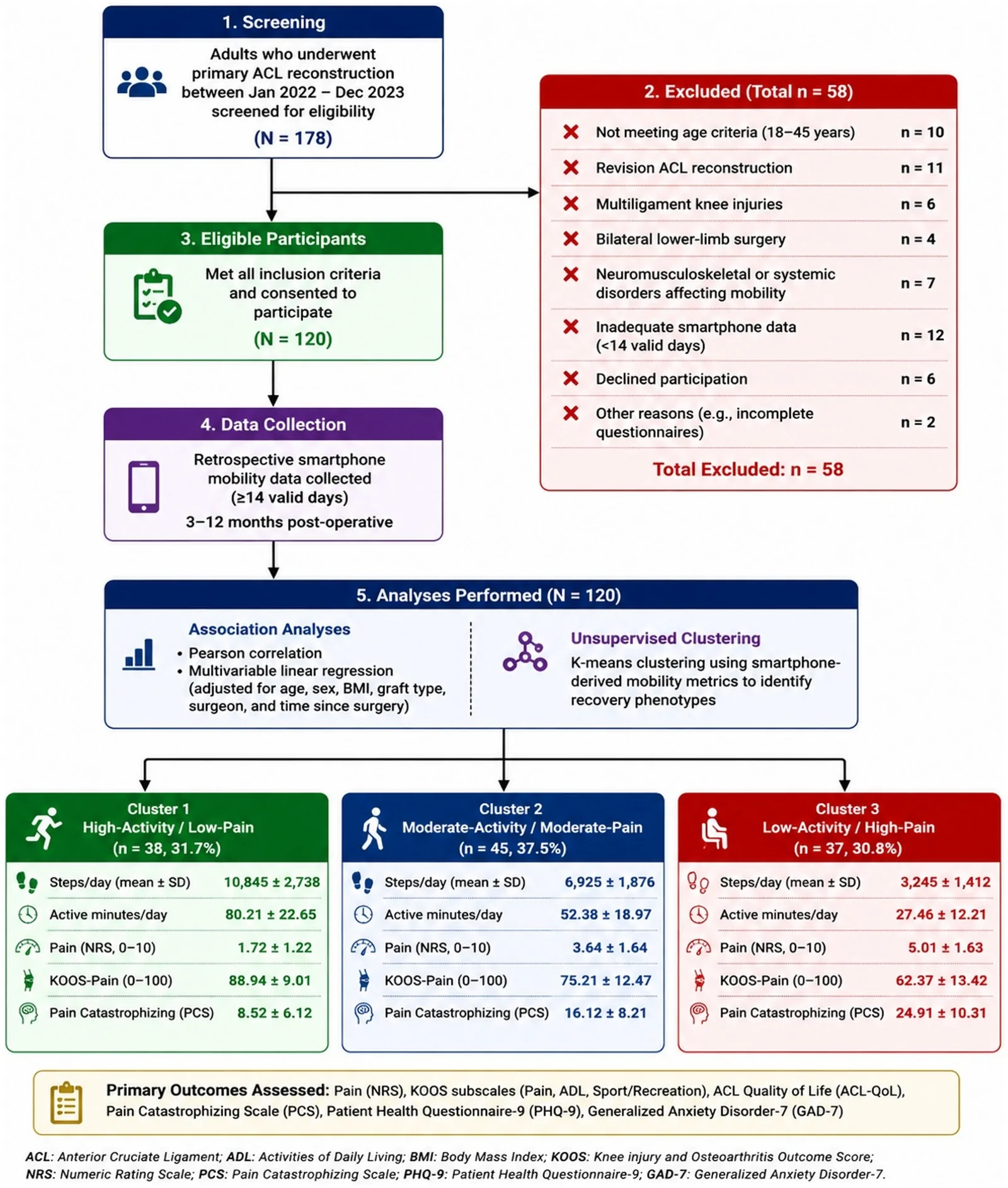
Participant selection flow diagram and mobility-based recovery phenotype classification following anterior cruciate ligament reconstruction

Participants were required to own a compatible smartphone (iOS or Android) with a native health application and to provide at least 14 consecutive days of valid mobility monitoring data, defined as ≥ 12 h of daily phone carry time in the 30 days preceding the study assessment. Additional exclusion criteria included bilateral lower-limb surgery, significant neuromusculoskeletal or systemic disorders affecting mobility, and inability or unwillingness to provide informed consent. Eligibility was verified by a research clinician before data extraction. A flow diagram summarizing participant screening, eligibility assessment, inclusion, and mobility-based phenotype classification is presented in Fig. 1. Postoperative rehabilitation followed institutional guidelines emphasizing pain and swelling control, restoration of knee extension, progressive range-of-motion exercises, quadriceps activation, weight-bearing progression, gait normalization, strengthening, and gradual introduction of running, pivoting, and sport-specific activities, although individual progression varied according to graft type, concomitant procedures, symptoms, and achievement of functional milestones.

Postoperative rehabilitation followed a standardized, criterion-based program delivered by licensed physiotherapists. During the early postoperative phase (0–2 weeks), rehabilitation emphasized pain and effusion control, restoration of full passive knee extension, progressive improvement in knee flexion, quadriceps activation, patellar mobilization, and gait retraining with progressive weight bearing as tolerated. Progression to the subsequent phase required satisfactory pain and swelling control, independent ambulation, and adequate quadriceps activation with near-complete knee extension. During the intermediate phase (2–6 weeks), rehabilitation focused on restoration of full range of motion, progressive lower-limb strengthening, closed-chain strengthening exercises, balance training, and neuromuscular control. Advancement was based on restoration of functional range of motion, minimal joint effusion, and the ability to perform strengthening exercises with appropriate movement quality. Between 6 and 12 weeks, strengthening intensity was progressively increased while proprioceptive, single-leg balance, and functional movement exercises were introduced. Low-impact cardiovascular exercise and sport-specific movement preparation were incorporated according to individual recovery. Beyond 3 months, rehabilitation emphasized advanced strengthening, plyometric training, agility, running progression, and sport-specific drills when clinically appropriate. Progression throughout rehabilitation was guided by achievement of predefined functional milestones rather than postoperative time alone, including adequate strength, absence of significant pain or effusion, satisfactory neuromuscular control, and acceptable performance on functional tasks. Although rehabilitation followed this standardized framework, exercise progression and treatment frequency were individualized by the treating physiotherapist according to surgeon recommendations and each participant’s clinical presentation.

Baseline assessment consisted exclusively of validated patient-reported outcome measures, including the KOOS Pain, ADL, and Sport/Recreation subscales and the ACL-QoL questionnaire; no performance-based functional testing or instrumented biomechanical assessments were conducted. The study employed a cross-sectional observational design, with participants assessed during routine clinical follow-up rather than at fixed postoperative intervals. Because the recovery stage may influence both mobility behavior and patient-reported outcomes, postoperative duration was recorded as a continuous variable and incorporated into all adjusted statistical models as a potential confounder. The inclusion of participants across different recovery stages facilitated the identification of mobility-based recovery phenotypes while accounting for the influence of time since surgery in subsequent analyses.

### Pain intensity

Pain intensity was measured using the 11-point Numeric Rating Scale (NRS) [14], which has been widely validated for musculoskeletal and orthopedic populations. Participants were asked to rate their average level of knee pain experienced over the previous seven days on a scale from 0 to 10, where 0 represents “no pain” and 10 represents the “worst imaginable pain.” The NRS was administered electronically via a self-completed survey at the study visit [14]. This tool was chosen due to its ease of use, high test–retest reliability, and established sensitivity to clinical change [15]. NRS scores were treated as continuous variables in statistical analyses, with higher scores indicating greater pain intensity.

### Functional status

Participants completed the full KOOS; analyses focused a priori on KOOS-Pain, KOOS-ADL, and KOOS-Sport/Recreation as functional recovery indicators, while knee-related quality of life was captured using the ACL-QoL [16]. Each KOOS subscale consists of multiple items scored on a 5-point Likert scale, which are then converted to a 0–100 score, with higher values indicating better function and lower symptom burden [16]. Participants completed the KOOS through a secure, electronic interface during the study visit. The KOOS has demonstrated high internal consistency (Cronbach’s *α* > 0.80) and construct validity in postoperative knee surgery populations [16]. Raw responses were processed and converted into subscale scores using standardized KOOS scoring algorithms.

### Knee-related quality of life

Knee-specific quality of life was evaluated using the ACL Quality of Life questionnaire (ACL-QoL), a validated 32-item instrument specifically developed for individuals with ACL injury or reconstruction [17]. The ACL-QoL captures multiple dimensions of knee-related well-being, including symptoms, activity limitations, emotional impact, and lifestyle adaptation [17]. Each item is scored on a 100-mm visual analog scale, and the total score is calculated as the mean of all items, yielding a final range of 0–100, with higher scores reflecting better quality of life [17]. Participants completed the questionnaire electronically during their follow-up visit, and item-level responses were checked for completeness before inclusion in the dataset [17].

### Smartphone-derived mobility metrics

Smartphone-based mobility metrics served as the primary independent variables [18]. Data were extracted retrospectively from participants’ native health-tracking applications (Apple Health for iOS, Google Fit, or Samsung Health for Android) covering at least 14 consecutive days within the 30 days prior to the study visit. To ensure data validity and completeness, only participants with at least 14 consecutive days of monitoring and a minimum average daily phone carry time of ≥ 12 h were included. Days not meeting this wear-time criterion were excluded from analysis. Because native health platforms may integrate data from synchronized wearable devices, step sources were reviewed during extraction. Only mobility variables recorded by the smartphone were retained; entries attributable to synchronized wearables were excluded when identifiable to reduce heterogeneity in measurement sources. Data quality indicators, including the number of valid monitoring days and the average daily phone carry time, were retained to assess data reliability and reduce bias from periods when the smartphone was not carried. Data synchronized from external wearable devices (smartwatches or third-party fitness trackers) were excluded when identifiable to minimize heterogeneity in measurement sources. Participants were instructed to confirm that their step data reflected smartphone-derived recordings rather than exclusively wearable-derived data. Extracted variables included average daily step count, standard deviation of daily steps (to reflect variability), average number and duration of walking bouts per day, total daily ambulatory distance (in kilometers), as recorded by the native smartphone health application (inclusive of walking and incidental running), and average active minutes per day.

Sedentary behavior was operationalized as the longest sedentary bout per day (defined as the maximum continuous inactivity recorded by the smartphone health platform), rather than total accumulated sedentary time. Data completeness was assessed using daily phone carry time and the number of valid monitoring days (≥ 12 h of use/day). The proportion of days achieving ≥ 6000 steps, a clinically meaningful physical activity threshold associated with functional recovery, was also calculated, consistent with thresholds established in prior rehabilitation research [21]. The ≥ 6000 steps/day threshold was selected as a pragmatic indicator of ambulatory recovery during the early-to-mid postoperative period and reflects step-count classifications commonly used to distinguish between lower- and moderately active adults in rehabilitation contexts. While ACL patients are often highly active preinjury, activity levels during the 3–12-month recovery phase remain variable and frequently below preinjury norms. Accordingly, this threshold was applied to stratify relative recovery status within the present cohort rather than to define an optimal or prescriptive activity target.

Daily step count, variability, and sedentary behavior metrics were calculated for each participant. Sensitivity analyses were additionally performed using stricter completeness thresholds to assess the robustness of clustering and regression results. Days with implausible values or incomplete recordings were excluded according to predefined criteria. Because passive smartphone monitoring relies on participants’ phone carriage during daily activities, data completeness and distribution were examined for irregular patterns suggestive of non-carry periods. Sensitivity analyses were conducted to evaluate whether excluding participants with lower monitoring consistency altered cluster structure or regression results.

### Pain catastrophizing

Pain-related cognitive distress was assessed using the Pain Catastrophizing Scale (PCS), a 13-item self-report questionnaire validated in orthopedic and chronic pain populations [19]. Each item is scored on a 5-point Likert scale ranging from 0 (“not at all”) to 4 (“all the time”), yielding a total score between 0 and 52. Higher scores represent more frequent and intense catastrophic thoughts related to pain, including rumination, magnification, and helplessness. The PCS was completed electronically at the study visit, and total scores were computed by summing item responses according to established scoring guidelines. This measure has demonstrated strong internal consistency and predictive validity for pain-related outcomes [20].

### Depression and anxiety

Psychological distress was evaluated using two validated screening instruments: the Patient Health Questionnaire−9 (PHQ-9) [21] for depressive symptoms and the Generalized Anxiety Disorder-7 (GAD-7) [22] for anxiety symptoms. Both instruments were administered via self-completed electronic surveys. The PHQ-9 consists of nine items, each scored 0–3, yielding a total score of 0–27, with a score ≥ 10 indicating moderate depression. Similarly, the GAD-7 includes seven items scored on the same 4-point scale, with total scores ranging from 0 to 21 and a cutoff of ≥ 10 indicating moderate anxiety. These instruments are widely used in orthopedic and rehabilitation research due to their diagnostic sensitivity and ease of administration. Because the PHQ-9 includes an item assessing suicidal ideation (item 9), predefined safety procedures were implemented. Surveys were administered within a supervised clinical setting, and responses indicating any level of suicidal ideation (score > 0 on item 9) triggered immediate notification of the supervising clinician. Participants were promptly evaluated and, when indicated, referred through established institutional mental health referral pathways. Data collection was conducted under the oversight of licensed healthcare professionals to ensure participant safety and appropriate clinical follow-up.

### Kinesiophobia (fear of movement)

Fear of movement or reinjury was measured using the Tampa Scale for Kinesiophobia-11 [23] (TSK-11), a shortened, validated version of the original 17-item TSK. The TSK-11 comprises 11 items, scored on a 4-point Likert scale from 1 (“strongly disagree”) to 4 (“strongly agree”), yielding a total score between 11 and 44. Higher scores indicate greater levels of movement-related fear [23]. Participants completed the TSK-11 electronically at the time of data collection, and total scores were calculated as the sum of all item responses. The TSK-11 has been shown to have strong internal consistency and validity in populations recovering from musculoskeletal injury [23].

All patient-reported outcome measures used in this study (NRS, KOOS, ACL-QoL, PCS, PHQ-9, GAD-7, and TSK-11) have been validated in musculoskeletal and postoperative orthopedic populations, including individuals in subacute and later phases of ACL rehabilitation. These instruments are appropriate for administration over the 3–12-month postoperative interval represented in the present cohort.

### Return-to-activity concerns

Perceived concern about returning to physical activity was assessed using a single-item numeric rating scale (NRS) developed for this study. Participants rated their level of concern about returning to activity on a scale from 0 (no concern) to 10 (extreme concern). This single-item measure was designed to capture perceived concern about returning to activity, rather than a comprehensive assessment. It was included as a brief exploratory indicator to minimize participant burden and was not intended to replace validated multi-item return-to-sport instruments such as the ACL Return to Sport after Injury (ACL-RSI) scale.

### Covariates

Demographic and surgical covariates included age, sex (male/female), body mass index (BMI), operative limb (right/left), graft type [bone–patellar tendon–bone (BTB) versus hamstring], time since surgery (in months), and presence of comorbidities (diabetes, hypertension). Comorbidities were defined as physician-diagnosed chronic medical conditions documented in the electronic health record and confirmed during clinical interview. These included metabolic disorders (diabetes mellitus), cardiovascular conditions (hypertension), respiratory disorders (asthma), and other chronic systemic conditions that could influence overall health status or activity levels but were not primary musculoskeletal exclusions. Smoking status was also recorded. These data were obtained from electronic medical records and confirmed during clinical interviews. All covariates were selected a priori based on their potential to confound associations between mobility behavior and recovery status, in accordance with prior literature on post-ACL reconstruction outcomes [24].

### Sample size calculation

Sample size estimation was performed a priori using G*Power 3.1.9.7 to determine the minimum number of participants needed to detect statistically significant associations between smartphone-derived mobility metrics and patient-reported outcomes in adults 3–12 months after ACL reconstruction. A multiple linear regression model with up to seven predictors was planned, incorporating both smartphone-derived variables (e.g., average daily steps, sedentary time, activity variability) and clinical covariates (age, sex, BMI, graft type, time since surgery). In practice, separate regression models were constructed for key mobility metrics to avoid overparameterization; each model included up to seven predictors entered simultaneously, comprising one primary smartphone-derived mobility variable and the predefined set of clinical covariates. This approach ensured consistency with the assumptions used in the a priori power calculation. Based on an assumed moderate effect size (f^2^ = 0.15), an alpha level of 0.05, and a desired statistical power of 0.90, the minimum required sample size was calculated to be 103 participants. To account for potential exclusions due to incomplete smartphone data or missing outcome measures, a 15% attrition rate was applied, resulting in a final target sample size of 120 participants.

### Data analysis

All analyses were performed using SPSS version 24.0 (IBM Corp., Armonk, NY, USA), with statistical significance set at a two-tailed *p*-value < 0.05. Continuous variables are presented as mean ± standard deviation, and categorical variables as frequency (percentage). Unsupervised clustering was used to identify mobility-based recovery phenotypes based on predefined smartphone-derived metrics, including average daily step count, step-count variability (standard deviation), average active minutes per day, longest sedentary bout duration, proportion of days achieving ≥ 6000 steps, average walking bout duration, and total ambulatory distance. Prior to clustering, mobility variables were screened for outliers and standardized to z-scores. Hierarchical agglomerative clustering using Ward’s method and squared Euclidean distance was initially performed to evaluate cluster structure. The optimal number of clusters was determined through dendrogram inspection, agglomeration coefficients, internal validation indices, and clinical interpretability. *K*-means clustering was subsequently applied to optimize cluster assignment and maximize between-cluster separation. Cluster stability was assessed through repeated resampling, assignment consistency, within- and between-cluster variance, and clinical coherence. Postoperative time since surgery was deliberately excluded from the clustering procedure to ensure that phenotypes reflected mobility behavior rather than the recovery stage. To evaluate the potential influence of postoperative timing, time since surgery was compared across clusters using appropriate between-group tests. Additional sensitivity analyses adjusted for postoperative month as a continuous variable in models examining associations between cluster membership and patient-reported outcomes. Between-cluster differences were evaluated using one-way analysis of variance (ANOVA) for continuous variables and chi-squared tests for categorical variables. When omnibus tests were significant, pairwise comparisons (cluster 1 versus cluster 2, cluster 1 versus cluster 3, and cluster 2 versus cluster 3) were conducted using Tukey’s Honest Significant Difference (HSD) test for continuous variables and adjusted pairwise procedures for categorical outcomes. Pairwise *p*-values are reported after correction for multiple comparisons. Associations between smartphone-derived mobility metrics and patient-reported outcomes were examined using Pearson correlation coefficients and interpreted as weak (0.00–0.39), moderate (0.40–0.69), or strong (≥ 0.70), consistent with published recommendations. Both statistical significance and effect size were considered when interpreting correlations. Multiple linear regression analyses were performed to evaluate the independent associations of mobility metrics (e.g., daily step count, step-count variability, active minutes, and sedentary bout duration) with KOOS subscales, ACL-QoL scores, and pain NRS. Models were adjusted for age, sex, body mass index, graft type, operating surgeon, and time since surgery. Time since surgery was included as a continuous covariate to account for its potential influence on recovery, while the surgeon was included to reduce confounding related to inter-surgeon differences in technique and perioperative management. Additional sensitivity analyses adjusted for phone carry time and smartphone platform type, where available, and restricted analyses to narrower postoperative intervals to evaluate robustness. Regression assumptions were assessed using residual and Q–Q plots to verify normality and homoscedasticity. Multicollinearity was evaluated using variance inflation factors (VIFs), with values < 5 considered acceptable. Missing data were assessed prior to analysis; a complete-case analysis was used because participants with incomplete mobility monitoring or outcome data were excluded during eligibility screening.

## Results

Participants were assessed at a mean of 7.2 ± 2.8 months following ACL reconstruction and generally demonstrated favorable functional recovery with high KOOS-ADL (84.6 ± 12.7) and ACL-QoL (71.8 ± 15.4) scores, accompanied by relatively low pain (NRS 2.8 ± 1.9) and mild psychological symptom burden (Table 1). Daily mobility averaged 6,825 ± 2,145 steps and 103.4 ± 34.2 active minutes, while hamstring tendon autografts were the most common graft type (55.5%); approximately half of participants had no concomitant meniscal pathology (49.2%), with meniscal repair being the most frequent additional procedure (28.1%).

**Table 1:** Baseline demographic, psychological, mobility monitoring, surgical, and concomitant pathology characteristics of participants following primary ACL reconstruction

Characteristic	Overall Cohort (*n* = 120)
Demographic characteristics	
Age, years	26.80 ± 7.10
Sex, female, *n* (%)	54 (42.2)
BMI, kg/m^2^	25.10 ± 3.80
Time since surgery, months	7.20 ± 2.80
Patient-reported outcomes	
KOOS-ADL	84.60 ± 12.70
ACL-QoL	71.80 ± 15.40
Pain NRS	2.80 ± 1.90
PCS	14.20 ± 8.10
PHQ-9	4.90 ± 3.80
GAD-7	4.30 ± 3.50
Monitoring characteristics	
Valid monitoring days	24.60 ± 4.80
Phone carry time, h/day	13.52 ± 2.31
Mobility metrics	
Average daily step count	6825 ± 2154
Average active minutes/day	52.38 ± 18.97
Maximum continuous inactivity, h/day	4.20 ± 1.50
Daily step variability (SD)	2148 ± 972
Surgical characteristics	
Hamstring tendon autograft, *n* (%)	71 (55.5)
Bone–patellar tendon–bone autograft, *n* (%)	43 (33.6)
Quadriceps tendon autograft, *n* (%)	14 (10.9)
Concomitant meniscal pathology	
None, *n* (%)	63 (49.2)
Medial tear, *n* (%)	29 (22.7)
Lateral tear, *n* (%)	24 (18.8)
Combined medial and lateral tear, *n* (%)	12 (9.4)
Meniscal treatment	
Meniscal repair, *n* (%)	36 (28.1)
Partial meniscectomy, *n* (%)	23 (18.0)
Meniscal repair + partial meniscectomy, *n* (%)	6 (4.7)
Concomitant chondral pathology	
Grade I–II lesion, *n* (%)	24 (18.8)
Grade III–IV lesion, *n* (%)	12 (9.4)
Cartilage procedures	
Chondroplasty, *n* (%)	9 (7.0)
Microfracture, *n* (%)	3 (2.3)

Data are presented as mean ± SD or *n* (%). Higher KOOS-ADL and ACL-QoL scores indicate better outcomes, whereas higher Pain NRS, PCS, PHQ-9, and GAD-7 scores indicate worse symptoms

*ACL* anterior cruciate ligament, *ACL-QoL* Anterior Cruciate Ligament Quality of Life, *BMI* body mass index, *GAD-7* Generalized Anxiety Disorder-7, *KOOS-ADL* Knee Injury and Osteoarthritis Outcome Score–Activities of Daily Living, *NRS* Numeric Rating Scale, *PCS* Pain Catastrophizing Scale, *PHQ-9* Patient Health Questionnaire-9, *SD* standard deviation

Smartphone-derived mobility monitoring indicated moderate activity levels with substantial inter-individual variability, supported by high data completeness over 27.5 ± 3.3 valid monitoring days and 13.5 ± 2.3 h/day of phone carry time (Table 2). Participants accumulated an average of 6825 ± 2,154 steps/day, with 72.5% achieving ≥ 6000 steps on most days. Sedentary behavior remained common, as evidenced by prolonged inactivity bouts (92.7 ± 31.7 min) and inactivity exceeding 30 min in 64.2% of participants. Activity patterns also varied across the week, with a mean weekend–weekday difference of 1032 ± 624 steps, indicating fluctuating mobility behavior.

**Table 2:** Summary of smartphone-derived mobility metrics (14–30 days prior to study visit)

Variable	Mean ± SD or % (*n*)
Average daily step count	6825.43 ± 2153.76
Standard deviation of daily steps	1852.67 ± 965.22
Median daily step count (IQR)	6550.00 (4800.00–8900.00)
Proportion of days ≥ 6000 steps	72.50% (87/120)
Average walking bouts per day	8.42 ± 2.31
Average walking bout duration (min)	5.61 ± 1.48
Average active minutes per day	52.38 ± 18.97
Maximum sedentary bout duration (min)	92.73 ± 31.65
Total walking/running distance per day (km)	5.04 ± 1.96
Step count variability (coefficient of variation)	0.31 ± 0.09
Proportion of days with > 30 min inactivity	64.17% (77/120)
Step count weekend versus weekday difference	1032.47 ± 624.38
Phone carry time per day (h)	13.52 ± 2.31
Days with valid data (≥ 12 h of phone use)	27.45 ± 3.28

*IQR* interquartile range, *SD* standard deviation, *km* kilometers, *min* minutes, *ACL* anterior cruciate ligament

**Fig. 2:**
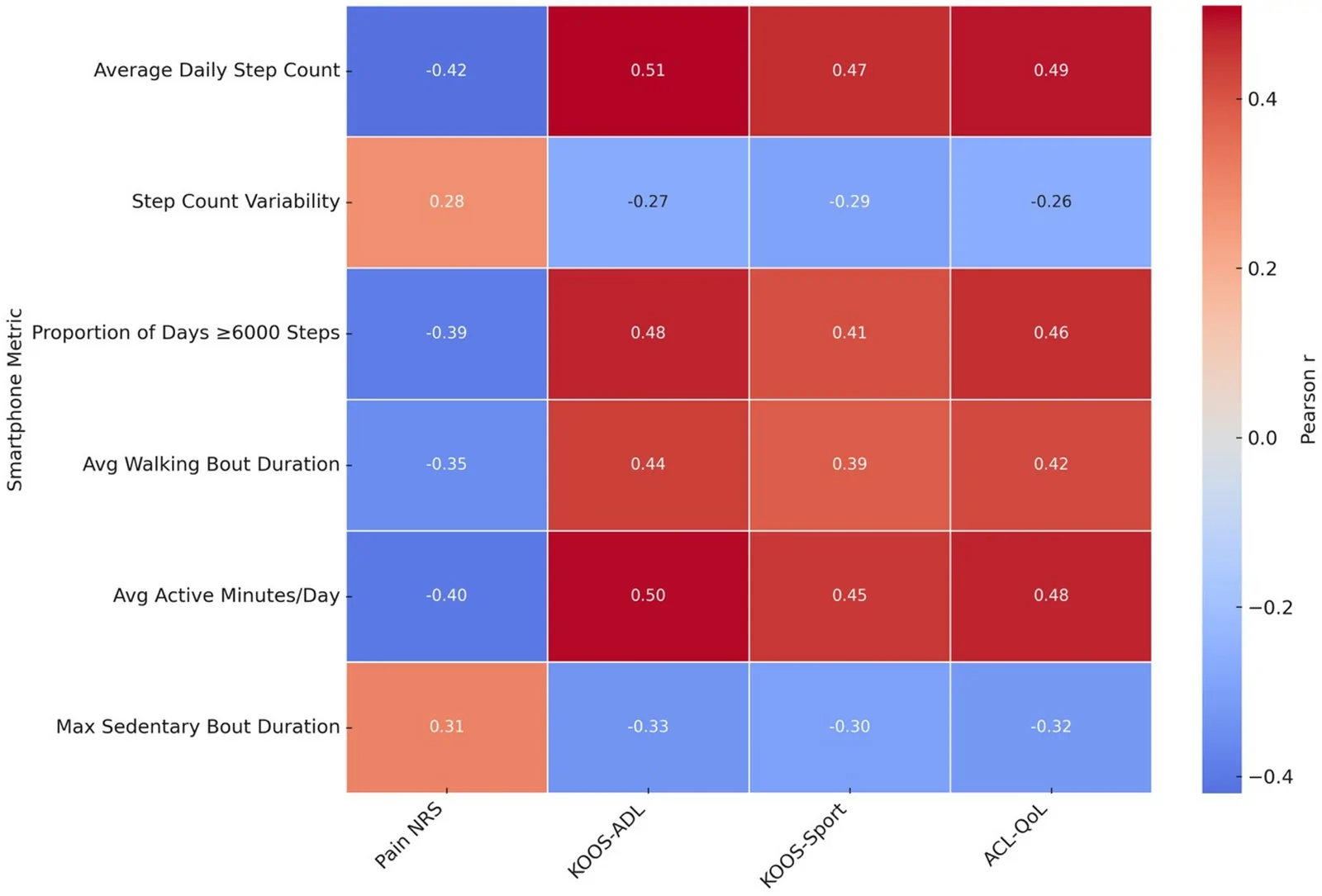
Correlations between smartphone-derived mobility metrics and patient-reported outcome measures

Higher levels of daily mobility were consistently associated with better patient-reported outcomes, whereas greater sedentary behavior and activity variability were associated with poorer recovery indicators (Fig. 2). Average daily step count and active minutes demonstrated the strongest moderate correlations with function, quality of life, and pain (*r* = 0.40–0.51), with step count showing the strongest association with KOOS-ADL (*r* = 0.51) and ACL-QoL (*r* = 0.49). In contrast, longer sedentary bouts and greater step variability were weakly to moderately associated with increased pain and worse functional outcomes (*r* = 0.26–0.33), highlighting the clinical relevance of sustained daily mobility patterns.

Higher daily mobility was independently associated with better postoperative recovery outcomes, with average daily step count emerging as the strongest predictor of function, quality of life, and pain (Table 3). Each additional 1000 steps/day was associated with higher KOOS-ADL (*β *= 2.38) and ACL-QoL (*β *= 3.06) scores and lower pain intensity (*β *= −0.31; all *p* < 0.001). Conversely, prolonged inactivity and greater pain catastrophizing were consistently associated with poorer outcomes, while time since surgery positively influenced recovery. The multivariable models demonstrated moderate predictive performance, explaining 42–49% of the variance in patient-reported outcomes (adjusted *R*^2^=0.42–0.49; all *p* < 0.001).

**Table 3:** Multivariable linear regression models predicting patient-reported outcomes from smartphone-derived mobility metrics after anterior cruciate ligament reconstruction

Predictor	Unit / reference	KOOS-ADL *β* (95% CI)	*p*-value	ACL-QoL *β* (95% CI)	*p*-value	Pain NRS *β* (95% CI)	*p*-value
Average daily step count	Per 1000 steps/day	2.38 (1.41–3.35)	< 0.001	3.06 (1.92–4.20)	<0.001	−0.31 (−0.44 to −0.18)	< 0.001
Average active minutes	Per 10 min/day	1.12 (0.48–1.76)	0.001	1.41 (0.66–2.16)	<0.001	−0.14 (−0.23 to −0.05)	0.002
Maximum continuous inactivity	Per 1 h/day	−1.45 (−2.26 to −0.64)	0.001	−1.74 (−2.69 to −0.79)	< 0.001	0.18 (0.07–0.29)	0.001
Daily step variability	Per 1000 steps SD	−0.68 (−1.29 to −0.07)	0.029	−0.83 (−1.54 to −0.12)	0.022	0.08 (−0.01 to 0.17)	0.083
Valid monitoring days	Per 1 day	0.21 (−0.06 to 0.48)	0.128	0.29 (−0.03 to 0.61)	0.074	−0.03 (−0.07 to 0.01)	0.143
Phone carry time	Per 1 h/day	0.62 (0.21–1.03)	0.003	0.74 (0.26–1.22)	0.003	−0.07 (−0.13 to −0.01)	0.018
Age	Per 1 year	−0.09 (−0.20 to 0.02)	0.108	−0.12 (−0.25 to 0.01)	0.071	0.01 (−0.01 to 0.02)	0.219
Sex	Female versus male	−1.86 (−4.38 to 0.66)	0.147	−2.42 (−5.38 to 0.54)	0.109	0.23 (−0.11 to 0.57)	0.184
BMI	Per 1 kg/m^2^	−0.31 (−0.62 to 0.00)	0.051	−0.36 (−0.72 to 0.00)	0.048	0.04 (0.00–0.08)	0.044
Graft type	BTB autograft versus hamstring autograft	−0.74 (−3.91 to 2.43)	0.646	−0.93 (−4.65 to 2.79)	0.623	0.12 (−0.31 to 0.55)	0.586
Concomitant meniscal procedure	Yes versus no	−1.31 (−3.98 to 1.36)	0.335	−1.68 (−4.82 to 1.46)	0.293	0.16 (−0.21 to 0.53)	0.394
Time since surgery	Per 1 month	0.44 (0.18–0.70)	0.001	0.52 (0.21–0.83)	0.001	−0.05 (−0.09 to -0.01)	0.011
Pain catastrophizing	Per 5 PCS points	−1.08 (−1.68 to −0.48)	< 0.001	−1.56 (−2.26 to −0.86)	< 0.001	0.19 (0.11–0.27)	< 0.001
Model adjusted *R*^2^	Overall model	0.46		0.49		0.42	
Model *F*-statistic	Overall model	10.84	< 0.001	12.36	< 0.001	9.58	< 0.001

*ACL-QoL* Anterior Cruciate Ligament Quality of Life, *ADL* activities of daily living, *BMI* body mass index, *CI* confidence interval, *KOOS* Knee injury and Osteoarthritis Outcome Score, *NRS* Numeric Rating Scale, *PCS* Pain Catastrophizing Scale, *SD* standard deviation.

Statistical note: regression coefficients are unstandardized *β* estimates from multivariable linear regression models adjusted for all predictors listed in the table. Higher KOOS-ADL and ACL-QoL scores indicate better outcomes, whereas higher Pain NRS scores indicate greater pain intensity. Adjusted *R*^2^ reflects the proportion of variance explained after accounting for the number of predictors.

**Fig. 3:**
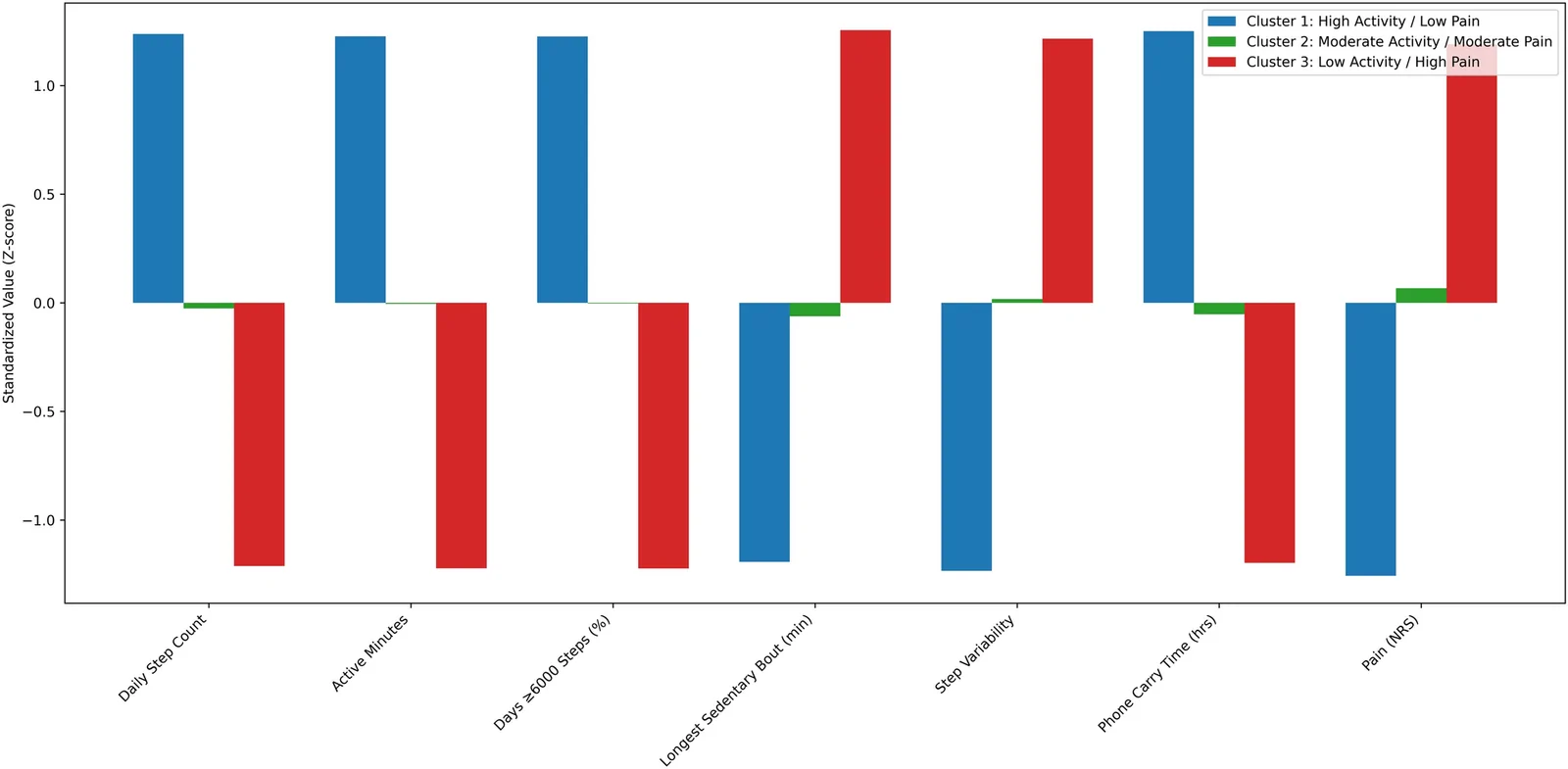
Standardized differences in smartphone-derived mobility metrics and pain across recovery phenotypes

Distinct mobility-based recovery phenotypes differed significantly in activity levels, sedentary behavior, and monitoring characteristics, supporting heterogeneous recovery patterns following ACL reconstruction (Fig. 3). Participants in the high-activity cluster demonstrated greater physical activity and less sedentary behavior than those in the low-activity cluster (all *p* < 0.001), whereas cluster membership was independent of graft type and was not fully explained by postoperative timing.

**Fig. 4:**
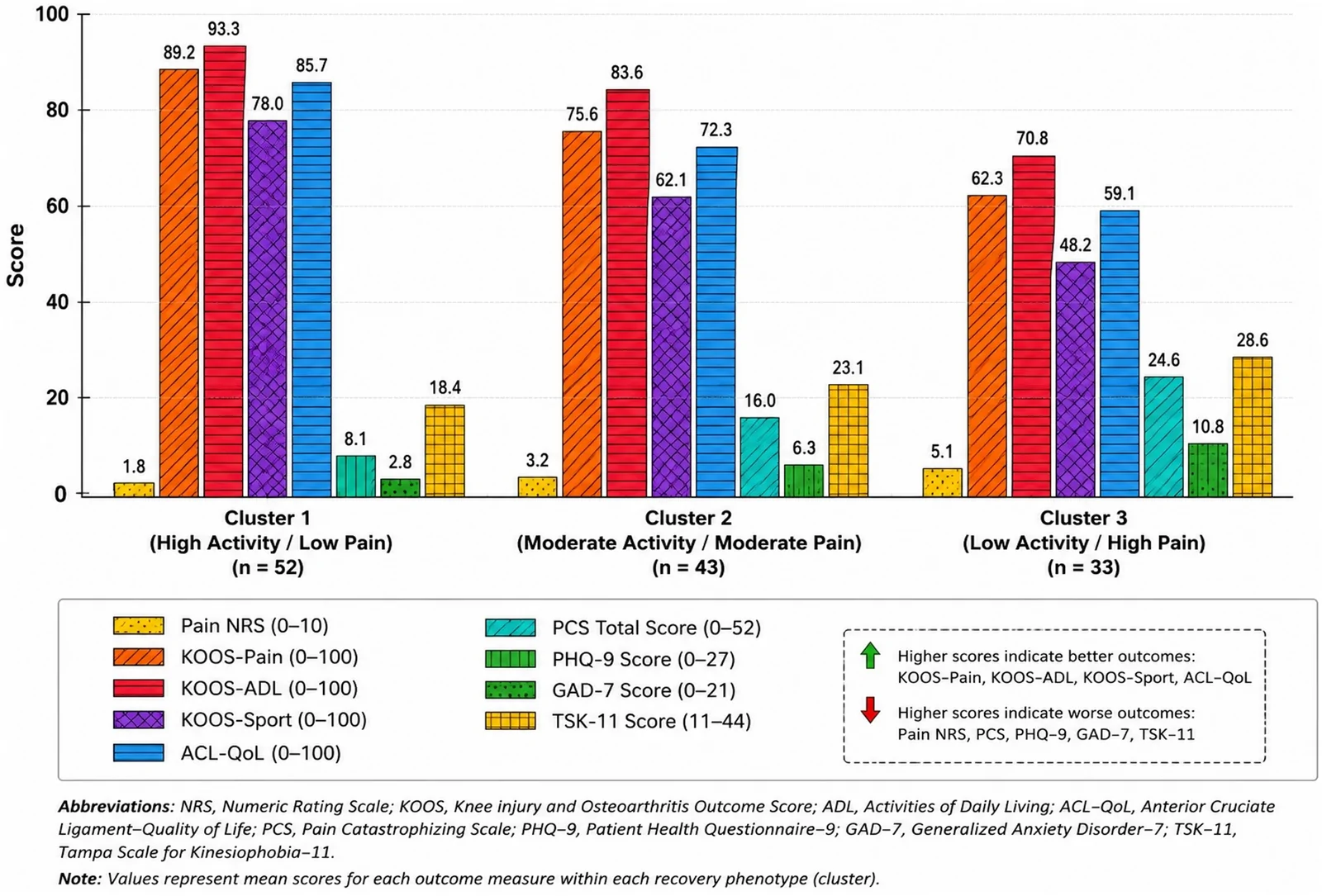
Patient-reported outcomes and psychological measures across smartphone-derived recovery phenotypes

Functional outcomes, pain, and pain catastrophizing demonstrated clear stepwise deterioration across the three mobility-based recovery phenotypes, supporting the clinical relevance of the clustering approach (Fig. 4). The low-activity/high-pain cluster exhibited substantially worse pain (NRS 5.01 versus 1.72), lower KOOS-Pain (62.37 versus 88.94), lower KOOS-ADL (71.04 versus 92.10), and poorer ACL-QoL (58.92 versus 85.13) compared with the high-activity/low-pain cluster (all *p* < 0.001). Pain catastrophizing also increased markedly across phenotypes (PCS 8.52–24.91; *p* < 0.001), whereas depressive and anxiety symptoms were descriptively higher in the low-activity group but did not differ significantly between clusters.

Marked differences in rehabilitation status and perceived recovery needs were observed across recovery phenotypes, indicating progressively greater clinical support requirements from the high-activity to low-activity groups (Table 4). Participants in the low-activity/high-pain cluster were substantially more likely to be receiving physiotherapy (64.9% versus 15.8%) and report unmet rehabilitation needs (56.8% versus 5.3%) than those in the high-activity/low-pain cluster (both *p* < 0.001). Fear of movement also increased significantly across phenotypes (*p* < 0.001), whereas depressive (PHQ-9) and anxiety (GAD-7) symptoms did not differ significantly between groups.

**Table 4:** Rehabilitation utilization and perceived needs across smartphone-derived recovery phenotypes

Variable	Cluster 1: high activity/low pain (*n* = 38)	Cluster 2: moderate activity/moderate pain (*n* = 45)	Cluster 3: low activity/high pain (*n* = 37)	*p*-value
Currently in physiotherapy (*n*, %)	6 (15.79%)	18 (40.00%)	24 (64.86%)	< 0.001
Discharged from physiotherapy (*n*, %)	32 (84.21%)	27 (60.00%)	13 (35.14%)	< 0.001
Self-reported unmet rehab needs (*n*, %)	2 (5.26%)	12 (26.67%)	21 (56.76%)	< 0.001
Fear of movement (TSK-11 score)	18.35 ± 4.21	23.44 ± 5.96	28.81 ± 6.48	< 0.001
Concern about returning to activity (0–10)	2.87 ± 1.48	4.94 ± 2.13	6.76 ± 2.42	< 0.001

*TSK-11* Tampa Scale for Kinesiophobia-11, *SD* standard deviation

## Discussion

This study examined associations between passively collected smartphone-derived mobility metrics and patient-reported outcomes following ACL reconstruction and identified distinct recovery phenotypes using unsupervised clustering. Higher levels of mobility, including greater daily step counts, active minutes, and favorable walking patterns, were associated with better function, lower pain, and improved quality of life. The identified phenotypes differed meaningfully in physical function, kinesiophobia, rehabilitation utilization, perceived recovery needs, and concern about returning to activity. Cluster membership was not explained solely by graft type or postoperative timing, suggesting that smartphone-derived mobility data capture important inter-individual differences in recovery beyond surgical and chronological factors. As this cross-sectional study cannot establish causality, the phenotypes should be interpreted as behavioral mobility profiles rather than longitudinal recovery trajectories. These findings support the potential value of digital mobility monitoring for personalized recovery assessment and postoperative care.

This study demonstrated robust associations between smartphone-derived mobility metrics and patient-reported outcomes among individuals recovering from ACL reconstruction. It is important to note that the magnitude of the regression coefficients was small for each 1000-step increment. Although statistically significant, such incremental changes are unlikely to represent clinically meaningful improvements in isolation. However, the between-cluster differences observed in daily step count exceeded 4000 steps/day, suggesting that broader differences in habitual mobility may translate into more meaningful functional and symptomatic variation. These findings therefore support a dose–response relationship rather than a discrete clinical threshold effect. Higher levels of objectively recorded physical activity, including step count and active minutes, were consistently associated with better self-reported function, lower pain, and enhanced quality of life [25]. These findings are supported by previous studies showing that digital mobility measures reflect meaningful aspects of recovery in musculoskeletal populations [11]. Crozat et al. [26] and Constantinescu et al. [7] findings align with evidence that wearable- and smartphone-derived activity metrics track recovery-relevant function in orthopedic populations. Crozat et al. [26], in a cohort of individuals with neuromuscular disorders, demonstrated that step-based metrics captured real-world functional changes not always detectable during clinic-based assessments. Additionally, indicators of sedentary behavior, such as prolonged periods of inactivity, were associated with poorer outcomes, corroborating the findings of Cardona et al. [27], who emphasized the detrimental effects of inactivity following orthopedic procedures. The present results support the clinical validity of passive smartphone monitoring as a scalable, patient-centered tool for tracking functional recovery beyond episodic in-clinic assessments.

Unsupervised clustering of mobility data revealed distinct recovery phenotypes that mapped onto meaningful differences in physical function, psychological status, and rehabilitation engagement [28]. Participants classified within the high-activity group consistently exhibited better pain and function scores, lower psychological distress, and fewer unmet rehabilitation needs, while those in the low-activity group presented with elevated levels of pain catastrophizing, kinesiophobia, and concern about return to activity [29]. These findings mirror previous research by Tan et al. [11], who identified behavioral phenotypes using mobile sensor data, and by Kesaria et al. [30], who reported that low-mobility profiles predicted prolonged rehabilitation needs and worse mental health outcomes. The current analysis reinforces the concept that real-world mobility phenotypes not only characterize physical capacity but also reveal psychological and behavioral dimensions of recovery [11]. This multidimensional profiling highlights the potential of smartphone-based monitoring to guide stratified, needs-based rehabilitation approaches in orthopedic care.

Differences in phone carry time were also observed across phenotypes and should be considered when interpreting mobility-based comparisons. Participants in the lower-activity phenotype demonstrated fewer hours of daily phone carry time, which may have reduced the opportunity for mobility events to be captured by the smartphone monitoring platform. Although phone carry time was included as a monitoring characteristic and differed significantly across clusters, residual measurement bias cannot be excluded. Consequently, some of the observed differences in mobility metrics may reflect variations in data capture rather than true differences in physical activity behavior. Future studies should investigate the influence of phone carry time and other indicators of data completeness on phenotype classification and mobility-based outcome assessment.

## Clinical significance

This study provides evidence that smartphone-derived mobility metrics, such as daily step count, active minutes, and sedentary patterns, are valid indicators of functional status, pain, and quality of life following ACL reconstruction. These digital measures offer clinicians an accessible, passive, and ecologically valid method to monitor real-world recovery between clinic visits. Importantly, the identification of distinct recovery phenotypes based on mobility profiles highlights the feasibility of stratifying patients into clinically meaningful subgroups with differing levels of physical function, psychological burden, and rehabilitation needs. Integrating such digital phenotyping into postoperative care could enhance the precision of follow-up strategies, support early identification of patients at risk of suboptimal recovery, and inform timely, tailored interventions without increasing patient or provider burden.

## Limitations and areas for future research

The study was limited by the retrospective, cross-sectional design, which precludes causal inference and introduces potential selection and recall bias. Smartphone-derived mobility metrics depended on consistent phone carriage and platform-specific settings, creating the possibility of measurement error despite predefined data-quality thresholds. A major methodological weakness was the broad and uncontrolled postoperative assessment window (3–12 months), which likely introduced substantial heterogeneity in recovery stage, rehabilitation progression, activity behavior, and symptom status that could not be fully addressed through statistical adjustment. Because recovery trajectories differ substantially between 3, 6, 9, and 12 months after ACL reconstruction, residual confounding related to recovery stage may have persisted despite statistical adjustment for time since surgery. Consequently, the identified phenotypes may partially reflect differences in recovery stage rather than purely distinct behavioral recovery profiles. Additional sources of residual confounding included variation in surgeon-related factors, autograft selection, surgical technique, concomitant meniscal and chondral pathology, associated procedures, and postoperative rehabilitation protocols, all of which may influence early recovery outcomes. Psychological readiness was assessed using a single-item measure rather than a validated return-to-sport instrument, and the cohort was restricted to individuals with compatible smartphones and adequate monitoring data, potentially limiting generalizability. Although key surgical and clinical variables were documented and adjusted where possible, detailed subgroup analyses were not performed; therefore, the identified mobility phenotypes should be interpreted within the broader surgical and rehabilitation context. Future prospective longitudinal studies with standardized follow-up intervals, validated psychological assessments, and more uniform treatment pathways are needed to improve internal validity and clarify recovery trajectories following ACL reconstruction.

## Conclusions

Smartphone-derived mobility metrics were associated with patient-reported outcomes and identified distinct recovery phenotypes following ACL reconstruction. Higher levels of daily activity and lower inactivity were associated with better function, quality of life, and lower pain, supporting the potential value of passively collected mobility data as objective indicators of postoperative recovery status. Smartphone-derived mobility metrics may provide scalable, objective measures for monitoring recovery following ACL reconstruction. However, given the variability in evaluation periods, heterogeneity in concomitant pathology and surgical management, and uncontrolled surgical factors, future research employing standardized, long-term follow-ups is warranted.

## Data Availability

The raw data supporting the conclusions of this article will be made available by the authors, without undue reservation.

## Abbreviations

ACL: Anterior cruciate ligament
ACL-QoL: Anterior cruciate ligament quality of life
ADL: Activities of Daily Living
BTB: Bone–patellar tendon–bone
GAD-7: Generalized Anxiety Disorder-7
KOOS: Knee Injury and Osteoarthritis Outcome Score
NRS: Numeric Rating Scale
PCS: Pain Catastrophizing Scale
PHQ-9: Patient Health Questionnaire-9
PROMs: Patient-reported outcome measures
QoL: Quality of life
TSK-11: Tampa Scale for Kinesiophobia-11
